# Incurring detriments of unplanned readmission to the intensive care unit following surgery for brain metastasis

**DOI:** 10.1007/s10143-023-02066-5

**Published:** 2023-06-29

**Authors:** Justus August Schweppe, Anna-Laura Potthoff, Muriel Heimann, Stefan Felix Ehrentraut, Valeri Borger, Felix Lehmann, Christina Schaub, Christian Bode, Christian Putensen, Ulrich Herrlinger, Hartmut Vatter, Niklas Schäfer, Patrick Schuss, Matthias Schneider

**Affiliations:** 1 Department of Neurosurgery, University Hospital Bonn, Venusberg-Campus 1, 53127 Bonn, Germany; 2 Department of Anesthesiology and Intensive Care Medicine, University Hospital Bonn, Bonn, Germany; 3 Division of Clinical Neuro-Oncology, Department of Neurology, University Hospital Bonn, Bonn, Germany; 4 Current address: Department of Neurosurgery, BG Klinikum Unfallkrankenhaus Berlin gGmbH, Berlin, Germany

**Keywords:** Unplanned ICU readmission, Brain metastasis, Surgery

## Abstract

**Object:**

Postoperative intensive care unit (ICU) monitoring is a common regime after neurosurgical resection of brain metastasis (BM). In comparison, unplanned secondary readmission to the ICU after initial postoperative treatment course occurs in response to adverse events and might significantly impact patient prognosis. In the present study, we analyzed the potential prognostic implications of unplanned readmission to the ICU and aimed at identifying preoperatively collectable risk factors for the development of such adverse events.

**Methods:**

Between 2013 and 2018, 353 patients with BM had undergone BM resection at the authors’ institution. Secondary ICU admission was defined as any unplanned admission to the ICU during the initial hospital stay. A multivariable logistic regression analysis was performed to identify preoperatively identifiable risk factors for unplanned ICU readmission.

**Results:**

A total of 19 patients (5%) were readmitted to the ICU. Median overall survival (mOS) of patients with unplanned ICU readmission was 2 months (mo) compared to 13 mo for patients without secondary ICU admission (*p*<0.0001). Multivariable analysis identified “multiple BM” (*p*=0.02) and “preoperative CRP levels > 10 mg/dl” (*p*=0.01) as significant and independent predictors of secondary ICU admission.

**Conclusions:**

Unplanned ICU readmission following surgical therapy for BM is significantly related to poor OS. Furthermore, the present study identifies routinely collectable risk factors indicating patients that are at a high risk for unplanned ICU readmission after BM surgery.

## Introduction

Brain surgery is an integral therapeutic component in the management of patients diagnosed with systemic malignancies and ensuing brain metastases (BM) in order to establish a definite diagnosis, relief of symptoms (e.g., symptoms of intracranial pressure, neurological deficits, seizures), and prolonging life expectancy [1–3]. Yet, studies demonstrated that after a magnetic resonance imaging-confirmed resection of the gadolinium-enhancing tumor areas, there is a 50% likelihood of BM recurrence in the field of the surgical bed [4–6]. This risk is markedly reduced by postoperative adjuvant therapy, reaffirming the value of conservative treatment modalities for BM. However, adjuvant therapy modalities (e.g., whole brain radiation, intraoperative radiotherapy, immunotherapy, chemotherapy) may be associated with a secondary decrease in the patient’s quality of life and therefore require an appropriate physical constitution of the patient following neurosurgical resection [7–9]. Against this backdrop, the initial postoperative period emerges as a vulnerable time span as potential complications (e.g., postoperative unfavorable events, internal medicine obstacles, epileptic events) might significantly prolong or even prevent the (necessary and time sensitive) initiation of adjuvant treatment modalities [10]. Unplanned readmission to the intensive care unit (ICU) following initial postoperative ICU monitoring after elective BM resection may serve as an indicator variable of such adverse events [11, 12]. The conduct of active intensive care treatment is often considered a reason for postponing potentially debilitating adjuvant therapy in cancer patients [13].

In the present study, we investigated the incidence and preoperative identifiable risk factors of unplanned ICU readmission and analyzed the prognostic impact of such unfavorable events in patients that had undergone surgery for BM.

## Methods

### Patients

All patients aged ≥ 18 years (yrs) that had undergone surgery for BM at the neuro-oncology center of the University Hospital Bonn between 2013 and 2018 were collected. The study was conducted in accordance with the Declaration of Helsinki and the protocol was approved by the Ethics Committee of the University Hospital Bonn (No. 250/19). Informed consent was not sought as a retrospective study design was chosen.

Preoperative obtainable information including patient age, patient sex, radiological features, laboratory values, location of primary cancer, functional status at admission and during the course of treatment, and the circumstance of unplanned ICU readmission were collected and entered into a computerized database (SPSS, version 27, IBM Corp., Armonk, NY). The comorbidity burden was determined using the Charlson comorbidity index (CCI). The CCI was derived from medical chart reviews and administrative systems [14]. After age adjustment, patients with BM were divided into two groups with CCI < 10 and CCI ≥ 10. The Karnofsky Performance Score (KPS) was used to classify the patients according to their functional status at admission. Patients were evaluated at admission according to their clinical–functional constitution with KPS ≥ 70% or KPS < 70%, as described previously [15]. In terms of the classification of the American Society of Anesthesiologists (ASA), the patients studied were divided into two groups: preoperative ASA 1 or 2 versus preoperative ASA ≥ 3. WBC counts (normal range 3.9–10.2 g/l) were divided into two groups, ≤12 g/l and >12 g/l, and CRP (normal range 0–3 mg/L) was dichotomized into ≤10 mg/l and >10 mg/l groups as previously described [10].

Within the time span of 2013 to 2018, all patients that underwent craniotomy for BM resection were routinely admitted to the ICU for initial postoperative monitoring. Unplanned ICU readmission was defined as any secondary postoperative ICU admission of a patient who had already been transferred to intermediate care unit or normal wards after uneventful routine postoperative ICU monitoring during the same hospital stay. Standard care protocol after BM resection included routine monitoring on ICU and patients were transferred to intermediate care unit or general ward the day after surgery. Standard care protocol after BM resection included routine monitoring on ICU and patients were transferred to intermediate care unit or general ward the day after surgery. Postoperative management included oral dexamethasone as well as intravenous or subcutaneous DVT prophylaxis from the day after surgery.

OS was defined as the time period from the day of surgery for BM until death or last observation in case the date of death was not known.

### Statistics

Data analyses were performed using the computer software package SPSS (version 25, IBM Corp., Armonk, NY) and PRISM. Categorical variables were analyzed in contingency tables using Fisher’s exact test. The Mann-Whitney *U* test was chosen to compare continuous variables as the data were mostly not normally distributed. Overall survival (OS) was analyzed by the Kaplan-Meier method using the GraphPad Prism software for MacOS (Version 9.4.1, Graphpad Software, Inc., San Diego, California, USA). The Gehan-Breslow-Wilcoxon test was used to compare survival rates. A backward stepwise method was used to construct a multivariable logistic regression model in order to identify preoperatively collectable predictors for unplanned ICU readmission. Results with *p* < 0.05 were considered statistically significant. The radar plot was generated using R (Version 3.6.2, Vienna, Austria).

## Results

### Baseline characteristics

Between 2013 and 2018, 388 patients had undergone resection of BM at the neurosurgical department of the University Hospital Bonn. In regard of 35 patients with insufficient follow-up information, the final study cohort was made up of 353 patients with surgically treated BM. Median age was 64 years (IQR 56–73) with 173 female (49%) and 180 male patients (51%). Three hundred eleven of 350 patients (88%) exhibited a preoperative KPS ≥ 70. One hundred twelve of 350 patients (32%) suffered from multiple BM. Most commonly BM originated from lung cancer (*n*=153, 43%), followed by breast cancer (*n*=45, 13%) and melanoma (*n*=37, 10%).

Unplanned readmission to the ICU was present in 19 of 353 BM patients (5%). Patients with unplanned ICU readmission yielded a median time span of 3 days (IQR 1.5-3.5 days) between both ICU stays. 13 patients (4%) died within 30 days after BM resection. Median OS (mOS) for the entire study cohort was 13 months (mo) (95% confidence interval (CI) 10.3–15.7). Further details are given in Table 1.

**Table 1:** Baseline characteristics*

	*n=*353
Median age (yrs) (IQR)	64 (56–73)
Female sex	173 (49)
Multiple BM	112 (32)
Preoperative KPS ≥ 70	311 (88)
Median age-adjusted CCI (IQR)	11 (10–12)
ASA ≥ 3	193 (55)
Primary site of cancer	
Lung	153 (43)
Breast	45 (13)
Melanoma	37 (10)
Others	118 (34)
Unplanned ICU readmission	19 (5)
Median time between ICU stays (days) (IQR)**	3 (1.5–3.5)
30-day mortality	13 (4)
Median OS (mo, 95% CI)	13 (10.3–15.7)

*Values represent number of patients unless indicated otherwise (%)

**For the group of patients with unplanned ICU readmission

*ASA*, American Society of Anesthesiology physical status classification system; *BM*, brain metastasis; *CCI*, Charlson comorbidity index; *IQR*, interquartile range; *KPS*, Karnofsky performance status; mo, months; *yrs*, years

### Reasons for unplanned ICU readmission

Reasons for unplanned postoperative ICU readmission in the present patient cohort were: postoperative hemorrhage (6/19, 31%) (resulting from therapeutic anticoagulation after pulmonary embolism (2/19, 11%), resulting from removal of the external intraventicular periprocedural drainage after resection of an infratentorial BM (1/19, 5%), resulting from secondary bleeding into the resection cavity (3/19, 16%)), neurologic deterioration (4/19, 21%) (resulting from postoperative impaired deglutition function (2/19, 11%), resulting from postoperatively worsened neurological morbidity due to postoperatively progressive edema (2/19, 11%)), respiratory failure (4/19, 21%), cardiovascular instability (2/19, 11%), intestinal perforation (1/19, 5%) (resulting from mechanical ileus due to intraabdominal tumor burden) and others (2/19, 11%) (Table 2).

**Table 2:** Reasons for unplanned ICU readmission*

Reasons for unplanned ICU readmission	*n=*19
Postoperative hemorrhage	6 (31)
Neurologic deterioration	4 (21)
Respiratory failure	4 (21)
Cardiovascular instability	2 (11)
Intestinal perforation	1 (5)
Others	2 (11)

*Values represent number of patients unless indicated otherwise (%)

*ICU*, intensive care unit

### Patient- and disease-related characteristics dependent on the occurrence of unplanned ICU readmission

**Fig. 1:**
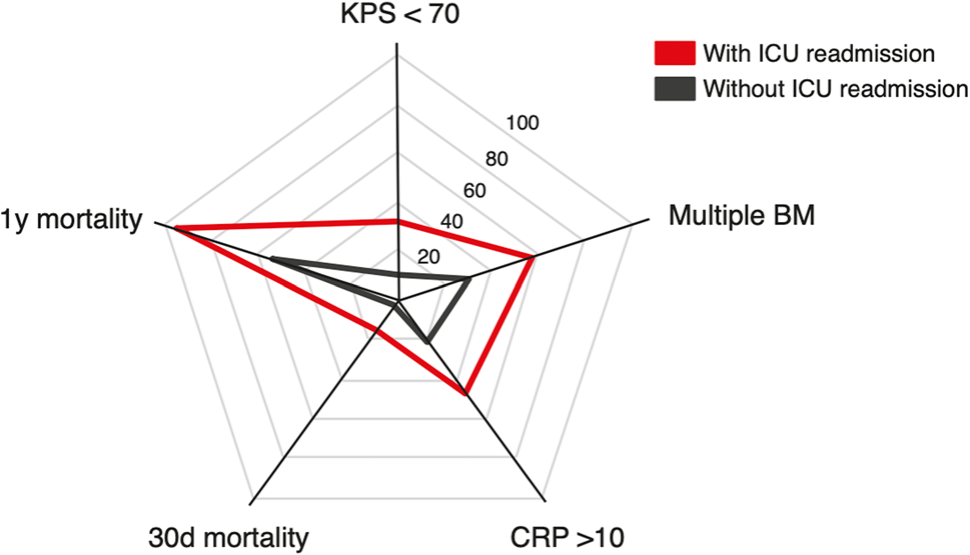
Radar plot depicting patient- and disease-related characteristics dependent on the occurrence of unplanned ICU readmission in patients with surgically treated BM. BM, brain metastasis; CCI, Charlson comorbidity index; CRP, C-reactive protein; d, day; ICU, intensive care unit; KPS, Karnofsky performance score; y, year

Patients with unplanned ICU readmission significantly more often revealed a preoperative KPS < 70 compared to patients without secondary ICU admission (37% vs. 11%, *p*=0.004) (Table 3, Fig. 1). Twelve of 19 patients (63%) with unplanned ICU readmission exhibited multiple intracranial BM compared to 100 of 334 patients (30%) without secondary ICU admission (*p*=0.004). Preoperative CRP > 10 mg/l was present in 9 of 19 patients (47%) with unplanned ICU readmission compared to 70 of 334 patients (21%) without unplanned ICU readmission (*p*=0.02). The groups of patients with and without unplanned ICU readmission did not significantly differ for both tumor volume, preoperative CCI values, ASA score and preoperative number of WBC, tumor entity and OP duration.

**Table 3:** Preoperatively identifiable patient and tumor related factors associated with unplanned ICU readmission*

	Patients without unplanned ICU readmission *n=*334	Patients with unplanned ICU readmission *n=*19	*p*-value
Median age (yrs, IQR)	65 (56–73)	63 (59–73)	0.7
Preoperative KPS < 70	35 (11)	7 (37)	**0.004**
Tumor volume (ml, IQR)	15 (6–25)	12 (8–25)	0.8
Multiple BM	100 (30)	12 (63)	**0.004**
CCI ≥ 10	247 (74)	15 (79)	0.8
ASA ≥ 3	181 (54)	12 (63)	0.5
Preoperative CRP > 10 mg/l	70 (21)	9 (47)	**0.02**
Preoperative WBC > 12 g/l	159 (48)	7 (37)	0.4
Primary site of cancer			
Lung	142 (43)	11 (58)	0.2
Breast	43 (13)	2 (11)	1.0
Melanoma	36 (11)	1 (5)	0.7
Others	113 (34)	5 (26)	0.6
Median OP duration (min, IQR)	169 (137–213)	188 (157–231)	0.3
30-day mortality	10 (3)	3 (16)	**0.03**
1-year mortality	186 (56)	18 (95)	**< 0.0001**
Median OS (mo, 95% CI)	13 (10.3–15.7)	2 (0.5–3.5)	**< 0.0001**

*Values represent number of patients unless indicated otherwise (%)

*BM*, brain metastasis; *ICU*, intensive care unit; *IQR*, interquartile range; *yrs*, years; *KPS*, Karnofsky Performance Scale; *BMI*, body mass index; *CCI*, Charlson comorbidity index; *ASA*, American Society of Anesthesiology; *OS*, overall survival, mo, months; *WBC*, white blood cells

**Fig. 2:**
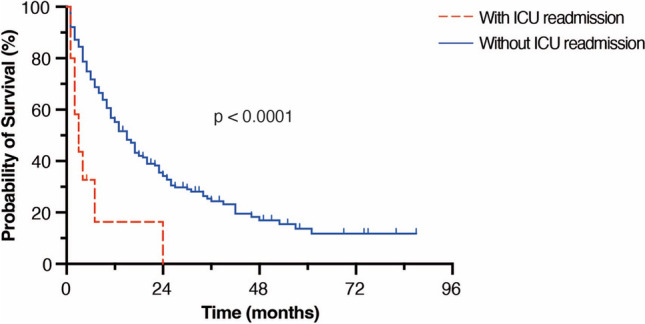
Kaplan-Meier survival curves depicting OS for patients with and without unplanned ICU-readmission. ICU, intensive care unit; OS, overall survival

Three of 19 patients (16%) with secondary ICU admission died within 30 days after surgery compared to 10 of 334 patients (3%) without unplanned ICU readmission (*p*=0.03). Unplanned ICU readmission was accompanied with significantly worsened mOS compared to a postoperative treatment course without unplanned ICU readmission (2 months vs. 13 months, *p*<0.0001) (Table 3, Fig. 2).

### Multivariable analysis identifies preoperative identifiable risk factors for unplanned ICU readmission in surgery for BM

We conducted a multivariable logistic regression analysis in order to identify preoperatively collectable risk factors for unplanned ICU readmission following resection of BM. The multivariate analysis identified “multiple BM” (*p*=0.02, OR 3.2, 95% CI 1.2–8.2) and “preoperative CRP levels > 10 mg/dl” (*p*=0.01, OR 3.2, 95% CI 1.3–8.5) as significant and independent predictors of unplanned ICU readmission (Nagelkerke’s *R*^2^ 0.1).

## Discussion

The present study was aimed at analyzing the incidence and patient- and treatment-related factors as well as the prognostic impact of secondary unplanned ICU readmission in cancer patients following resection of BM. Previous studies have evaluated the impact of ICU readmission and mortality in an unselected patient cohort of critically ill patients [16]. This is the first report on unplanned ICU readmission in a patient cohort of severe stages of systemic cancer that is cancer patients with BM undergoing neurosurgical resection.

In total, 5% of the patients with surgically resected BM were readmitted to the ICU. These observations are within the range of published readmission rates ranging from 3 to 17% for pooled surgical and medical ICU patients [17–20]. There is limited data on ICU readmission in oncohematological and thoracic oncological patients, indicating readmission rates up to 9% [21, 22]. Secondary ICU admission in the present series was accompanied by a significantly worsened mOS rate of 2 mo with only one patient who reached survival of more than 12 mo. This observation portends to the already highly vulnerable cohort of patients with BM where further unfavorable events impair initially intended surgical prognostic benefit. The 30-day mortality rate of 16% in the cohort of BM patients with secondary ICU admission is within the range of published mortality data for unselected critical ill patients that are readmitted to the ICU reaching up to as much as 30% [19].

The present study identified several patient- and disease-related factors that significantly correlated with an elevated risk for the need of a secondary ICU management. The patient group with multiple BM appeared to have a significantly higher probability to be readmitted to the ICU. Though the presence of multiple intracranial metastatic lesions generally is regarded as an independent predictor of poor prognosis [23, 15], there is growing literature supporting resective treatment modalities even at the stage of advanced systemic cancer with several BM. Peak et al. reviewed a series of patients with 2–3 BM and observed similar benefits of neurosurgical resection in this selected patient group compared to patients with solitary single BM [24]. Similarly, Bindal et al. reported beneficial survival data for the resection of several BM in selected patients with multiple BM compared to age-matched patients with single BM [25]. Up to date, the surgical management of patients with multiple BM, especially with more than 3 intracranial metastatic manifestations, remains controversial [26]. Either way optimal neurosurgical treatment management might crystallize in the future decades, the present series and previous literature indicate this patient clientele to be at a high risk for postoperative unfavorable events as indicated by the higher rate of unplanned readmission to the ICU.

We used laboratory parameters, obtained in routine preoperative blood diagnostics, for analysis of a potential prognostic impact for patients with BM. A level of CRP > 10 mg/L was strongly associated with an elevated probability of unplanned readmission to the ICU. CRP as an acute-phase protein increases in response to inflammation, trauma, and infection [27]. Systemic cancer is known to be associated with chronic inflammation signaling [28]. Elevated CRP levels have been linked especially to metastatic rather than non-metastatic cancer highlighting a particular importance of CRP as a systemic marker in advanced metastatic cancer [29, 30]. Non-small cell lung cancer patients with CRP levels higher than 40 mg/L were more likely to suffer from metastatic systemic cancer with a specificity of 100% [31]. Considering the existing literature, CRP appears to constitute a biomarker of growing importance for metastatic stages and survival in cancer patients [30]. The occurrence of increased adverse cardiovascular complications in the further course of treatment of these patients represents another possibility, as elevated CRP also seems to be associated with such a risk [32] which might partly provide a rationale for the link between elevated CRP levels and the risk for unplanned ICU readmission as seen in the present series.

It must be emphasized that the present study does not intend to restrict surgical therapeutic options in certain patients with BM. Rather, the authors aim at empowering a more comprehensive counseling of patients, family members, and caregivers based on the awareness of relevant preoperatively collectable risk factors associated with an increased risk of unplanned ICU admission following neurosurgical resection of BM. Furthermore, preoperative identification of patients at risk for the need of unplanned postoperative ICU management might facilitate more comprehensive postoperative monitoring and thus might contribute to the prevention of unplanned ICU readmission events. Given the heterogeneity of cancer patients with BM, further multicenter studies/registries are expected to be warranted in order to comprehensively explore the impact as well as potential risk factors for secondary unplanned ICU admission after surgery for BM.

### Limitations

The present study has several limitations. The study was conducted in a retrospective fashion and patients were not randomized, but treated according to the preferences of the treating physicians. Based on the retrospective data collection, a more specific determination of the underlying reasons for elevated CRP levels was beyond the scope of the present work. Furthermore, the patient group with unplanned ICU readmission was quite small and therefore hardly allowed any conclusions to be drawn about the underlying causes. Furthermore, the patient clientele with BM constitutes quite a heterogeneous study population in regard to the underlying cancer disease as well as pretreatment which might lead to relevant unmasked bias in data analysis. Nevertheless, the present study is the first to investigate unplanned ICU readmission in the course of surgery for BM and thus provides the basis for the initiation of further scientific pursuits.

## Conclusions

Unplanned ICU readmission following surgical therapy for BM is significantly related to poor overall survival. Furthermore, the present study identifies routinely collectable risk factors that may help to preoperatively detect patients who are at high risk for secondary ICU admission after BM surgery.

## Data Availability

Restrictions apply to the availability of these data due to privacy restrictions.
